# Nurses’ Experiences with Spiritual Care in Paediatric Palliative Care: A Systematic Review

**DOI:** 10.3390/healthcare14131994

**Published:** 2026-07-04

**Authors:** Sergej Kmetec, Anja Veber, Irena Maguša, Cvetka Krel, Nataša Mlinar Reljić

**Affiliations:** 1 Faculty of Health Sciences, University of Maribor, Žitna Ulica 15, 2000 Maribor, Slovenia; cvetka.krel@um.si (C.K.); natasa.mlinar@um.si (N.M.R.); 2 Division of Paediatrics, University Medical Centre Ljubljana, Zaloška Cesta 2, 1000 Ljubljana, Slovenia; anja.veber@gmail.com; 3 University Division of Gynaecology and Perinatology, University Medical Centre Maribor, Ljubljanska Ulica 5, 2000 Maribor, Slovenia; irena.magusa@ukc-mb.si

**Keywords:** child, paediatric palliative care, spiritual care, nurses, nursing experiences, end-of-life care, thematic synthesis, emotional burden

## Abstract

**Highlights:**

**What are the main findings?**

- Nurses in paediatric palliative care experience significant emotional and spiritual burdens, including guilt, helplessness, and anxiety, when caring for dying children and their families.
- A persistent gap exists between nurses’ recognition of the importance of spiritual care and their actual capacity to provide it, associated with insufficient education and limited institutional support.

**What are the implications of the main findings?**

- Targeted educational programmes in spiritual care competency and structured clinical supervision are urgently needed for nurses in paediatric palliative care.
- Institutional policies should formally address nurses’ psychosocial and bereavement support needs to prevent burnout and sustain compassionate practice.

**Abstract:**

**Background/Objectives**: Spiritual care is a core component of holistic paediatric palliative care, yet nurses often feel insufficiently prepared to address the spiritual and existential needs of seriously ill children and their families. This systematic review aimed to explore nurses’ experiences of providing spiritual care to seriously ill and dying children in paediatric palliative care settings and to identify the factors that facilitate or hinder its provision. **Methods**: A systematic review was conducted in accordance with PRISMA 2020. CINAHL, PubMed, Web of Science and SAGE were searched for English-language qualitative, quantitative and mixed-methods studies published up to November 2025. Study quality was assessed using JBI critical appraisal checklists, and the findings were synthesised thematically following Thomas and Harden. **Results**: A total of 228 records were identified, of which ten studies met the predefined inclusion criteria. The thematic synthesis identified one overarching theme—nurses’ engagement with spirituality while caring for seriously ill and dying children—supported by two sub-themes: managing emotional responses and maintaining professional, family-centred support. **Conclusions**: Nurses recognise spiritual care as essential in paediatric palliative care but often lack the competence and institutional support to provide it consistently. Education should prioritise spiritual assessment, developmentally appropriate communication, ethical boundaries, reflective practice and structured debriefing.

## 1. Introduction

Globally, an estimated 21 million children require some form of paediatric palliative care (PPC), with more than 8 million needing access to specialist services [1]. Nearly 2.5 million children die each year with serious health-related suffering, most of them in low- and middle-income countries [2]. Palliative care (PC) is defined by the World Health Organization (WHO) as a holistic approach that seeks to prevent and relieve physical, psychosocial, and spiritual distress in people facing life-limiting illness [3]. Within this framework, PPC has evolved into a distinct subspecialty focused on alleviating suffering and enhancing the quality of life for children and their families affected by progressive, life-limiting conditions [4].

Advances in medical treatment have extended the survival of children with complex chronic conditions, such as cancer, neurodegenerative disorders, metabolic diseases, and congenital anomalies. This progress has created new demands for continuous and integrated palliative care services [5,6,7]. Meeting these demands requires strategic planning, workforce development, and evidence-informed policy.

Despite global advocacy for holistic PPC, substantial inequities persist in access to services, quality of care, and professional capacity. Low- and middle-income countries bear the greatest burden of need yet have the least access to specialised PPC, reflecting disparities in training, resources, and policy prioritisation [1,2]. Even in high-income settings, spiritual and psychosocial care remain inconsistently integrated into paediatric practice models, often because of the dominance of biomedical frameworks and limited interprofessional collaboration [1]. A growing body of evidence underscores the importance of culturally sensitive, family-centred, and spiritually inclusive care frameworks, demonstrating that spiritual well-being contributes to quality of life, treatment adherence, and emotional resilience among children and families facing life-limiting illness [8].

Although physical symptom management is often the primary focus in PPC, spiritual care is a fundamental dimension of holistic support, fostering meaning, resilience, and emotional well-being in children and their families [8]. For this review, spirituality is understood as a dynamic and intrinsic aspect of humanity through which persons seek ultimate meaning, purpose, and transcendence and which may or may not be expressed through formal religious practice [9]. Spirituality is thus distinguished from religiosity, which refers specifically to adherence to organised religious traditions, and from existential care, which addresses questions of meaning, identity, and suffering within a secular framework. This conceptual distinction is particularly relevant, as the broader nursing and palliative care literature has not consistently applied these terms—a challenge further addressed in the Discussion. Nevertheless, spiritual needs are often overlooked, commonly due to insufficient clinician preparation and discomfort with addressing spiritual or existential concerns [10,11]. Nurses spend significant time with seriously ill children and are well-positioned to assess and respond to their multidimensional needs, provided they receive appropriate education and institutional support [12,13].

Despite this potential, integrating spiritual care into everyday nursing practice remains constrained by conceptual ambiguity, insufficient formal education, and a lack of practical guidance. Evidence indicates that nurses often equate spirituality with religiosity, creating uncertainty about how to assess and respond to diverse spiritual expressions in secular or pluralistic healthcare settings [14]. Additionally, time constraints, emotional fatigue, and institutional cultures that undervalue the non-physical dimensions of care further limit the provision of spiritual support [10,11]. These challenges underscore the need for clearer conceptual frameworks, validated assessment tools, and structured educational programmes that equip nurses to deliver evidence-based spiritual care within multidisciplinary PPC teams.

The Slovenian context presents similar challenges. Research from the University Children’s Hospital in Ljubljana indicates that many children requiring PPC are identified late and that services remain insufficiently integrated across levels of care [15]. Studies of Slovenian nursing practice further suggest that formal education on spirituality is limited and that confidence in delivering spiritual care varies with training exposure [16]. Strengthening competence in spiritual care is therefore a national priority, particularly as Slovenia continues to expand paediatric palliative initiatives. The Slovenian context is introduced here as a national example of broader European challenges in integrating spiritual care into nursing practice and as the motivating policy context for this review.

Despite the recognised importance of spiritual care in PPC, a comprehensive synthesis of nurses’ lived experiences in this domain remains scarce. Existing reviews have examined healthcare staff experiences at the end of life more broadly—most notably McConnell, Scott and Porter [17], who synthesised 35 studies across the multidisciplinary workforce. However, that review was not restricted to nurses, did not isolate spiritual care as a distinct domain, and did not employ a formal thematic synthesis. Reviews on spiritual care in nursing practice have likewise focused predominantly on adult palliative or general clinical settings. It is therefore essential to understand how nurses perceive, navigate, and are influenced by spiritual care provision specifically within PPC, along with the barriers and facilitators they encounter. This understanding will guide curriculum development, institutional policy, and evidence-based practice. This systematic review, therefore, aimed to explore nurses’ experiences of spiritual care in PPC and to identify the factors that influence their capacity to provide it.

## 2. Materials and Methods

This article presents a systematic review. The review protocol was prospectively registered in 2026 on the OSF Registries https://osf.io/y2ktm (accessed on 17 May 2026). The design, conduct, and reporting of the review followed the guidelines set forth by the Preferred Reporting Items for Systematic Reviews and Meta-Analyses 2020 (PRISMA 2020) [18].

### 2.1. Research Question

The research question was formulated using the Population, Interest, and Context (PICo) framework, as recommended by the JBI for qualitative evidence synthesis [19]. The components were defined as follows: Population (P)—registered nurses in paediatric palliative care settings; Interest (I)—nurses’ experiences of providing spiritual care to seriously ill and dying children and their families; and Context (Co)—paediatric palliative care, encompassing hospital-based, community, and home-based settings.

The research question was: “What experiences do nurses have with spiritual care in paediatric palliative care settings, and what factors facilitate or hinder its provision?”

### 2.2. Search Strategy

A comprehensive search strategy was developed using English-language synonyms for key concepts, combined with Boolean operators (AND, OR). The final search string was: (experience* OR perception) AND nurse AND (spiritual* OR “spiritual care”) AND (child* OR “young adult*” OR adolescent* OR youth) AND “paediatric palliative care”. The search was conducted across four electronic databases: CINAHL, PubMed, Web of Science, and SAGE. SAGE Journals was included because it indexes a range of nursing, palliative care, and health sciences journals not fully covered by PubMed, CINAHL, or Web of Science. However, SAGE does not support controlled vocabulary searching, and the broader keyword-based strategy accordingly yielded a high volume of records (*n* = 197) relative to the number ultimately included (*n* = 1). This low precision is acknowledged as a limitation of the SAGE search component; it reflects the platform’s structural constraints rather than an error in search design. The string presented above is a simplified, readable summary. The complete database-specific search strategies, adapted to the controlled vocabulary and field codes of each database (including MeSH terms for PubMed and CINAHL headings), are provided in full in Supplementary Material S1 and were used in practice.

In addition, a hand search of the grey literature was conducted, including the reference lists of all included studies and the websites of relevant international organisations: the World Health Organization, the International Children’s Palliative Care Network, the European Association for Palliative Care, the National Hospice and Palliative Care Organization, and the International Association for Hospice and Palliative Care. No additional records were identified through this process. The search was restricted to English-language studies published up to November 2025. All retrieved records were imported into reference management software (EndNote 20) for deduplication.

### 2.3. Eligibility Criteria

Eligibility criteria were defined prospectively in line with the PICo framework.

Inclusion criteria: (1) registered nurses or nursing students as primary study participants; (2) studies examining experiences, perceptions, or attitudes regarding spiritual care or spirituality in paediatric palliative care; (3) qualitative, quantitative, or mixed-methods study designs; (4) studies published in English; and (5) studies published up to November 2025.

Exclusion criteria: (1) studies not focused on spirituality or spiritual care as a primary focus; (2) studies conducted exclusively in adult or non-paediatric palliative care populations; (3) review articles, editorials, conference papers, and letters to the editor; and (4) studies with a JBI critical appraisal score below 70%. This threshold was selected based on precedent in comparable qualitative evidence syntheses and to ensure adequate reporting of methodological rigour, including researcher reflexivity and data saturation—both critical to the credibility of qualitative synthesis. Although JBI guidelines do not specify a specific numerical cut-off, the 70% threshold is widely used in systematic reviews that use JBI appraisal tools [20,21].

### 2.4. Study Selection

Records were screened in two sequential stages. Three authors (AV, SK, NMR) independently screened all titles and abstracts. Inter-rater reliability before consensus discussions was assessed using pairwise percentage agreement and Cohen’s kappa across the three reviewer pairs. Disagreements were resolved through discussion with a co-author (IM). Second, full-text articles that passed the title and abstract screening stage were retrieved and independently assessed against the eligibility criteria by the entire author team. Final inclusion decisions were made by consensus.

### 2.5. Critical Assessment

All studies meeting the inclusion criteria (Table 1) underwent critical appraisal using the JBI appraisal checklists appropriate to each study design. The JBI Qualitative Research Appraisal Checklist (10 items) [22] was used for qualitative studies, and the JBI Critical Appraisal Checklist for Analytical Cross-Sectional Studies (8 items) [23] was used for the quantitative study. Responses were scored as Yes (1 point), No (0 points), Unclear (0 points), or Not Applicable (0 points). A total quality score, expressed as a percentage, was calculated for each study. Studies were categorised as low quality (60–69%), moderate quality (70–79%), high quality (80–89%), or excellent quality (≥90%) [24]. A minimum score of 70% was required for inclusion in the review, consistent with established recommendations for qualitative systematic reviews [22]. Critical appraisal was independently conducted by three authors (AV, SK, NMR), and any discrepancies were resolved through discussion with the fourth author (IM) (Table 2).

Following the thematic synthesis, confidence in each synthesised finding was assessed using the GRADE-CERQual approach [25], which evaluates four components: methodological limitations of contributing studies, coherence of the synthesised finding, adequacy of data, and relevance to the review question. Assessments were conducted independently by two authors (SK, AV) and presented in a summary of qualitative findings table (Table 3).

The potential for reporting bias was assessed during the review process. As the search was restricted to published, English-language studies, studies with null, inconclusive, or negative findings may be underrepresented in the evidence base. Thesis databases, including ProQuest Dissertations & Theses Global and EThOS, and conference proceedings were not systematically searched, which represents a potential source of publication bias that may have led to underrepresentation of null or inconclusive findings. This limitation was taken into account when interpreting the synthesised findings.

### 2.6. Data Extraction and Synthesis of the Data

Data were extracted from all included studies using a standardised extraction form. Extracted items included the first author, year of publication, country, study aim, participant characteristics, research design, and key findings. Three authors (AV, SK, NMR) independently extracted the data, and discrepancies were resolved through discussion with the fourth author (IM) until consensus was reached.

Thematic synthesis was conducted following the three-stage method described by Thomas and Harden [26]. In the first stage, we carefully read the findings sections of all included studies line by line and generated free codes that captured the content and meaning of each finding. In the second stage, we grouped similar free codes and translated them into descriptive themes that accurately reflected the original studies’ content without exceeding it. In the third stage, we reviewed and interpreted the descriptive themes across the studies to develop analytical themes—higher-order interpretations that provide new insights into nurses’ experiences of spiritual care. These analytical themes comprise the overarching framework reported in Section 3. Three authors (AV, SK, NMR) independently conducted all three stages, and any discrepancies were discussed and resolved with the fourth author (IM).

As nine of the ten included studies employed qualitative designs, thematic synthesis was the primary analytical approach. The one quantitative study [27] was not amenable to statistical pooling; its findings were therefore integrated descriptively: quantitative results, including regression coefficients and predictor significance levels from Conner & Uddin [27], were interpreted narratively and mapped onto the emerging thematic framework at the stage of generating descriptive themes, where they served to contextualise and corroborate—rather than drive—the qualitative findings. The quantitative study did not contribute to theme generation but was used to confirm and extend the interpretive reach of themes already emerging from the qualitative synthesis. This approach is consistent with guidance for mixed-methods systematic reviews in which quantitative evidence is incorporated as supplementary contextual data rather than as the basis for meta-analysis [28].

## 3. Results

### 3.1. Study Identification and Selection

The electronic database search yielded 228 records: PubMed (*n* = 7), CINAHL (*n* = 17), Web of Science (*n* = 7), and SAGE (*n* = 197). After automated deduplication in EndNote 20, 210 unique records remained. Screening of titles and abstracts excluded 175 records that did not meet the predefined eligibility criteria. Thirty-five full-text articles were retrieved for detailed evaluation; of these, 25 were excluded after full-text review. Of the 25 full-text articles excluded, the reasons for exclusion were as follows: studies not conducted in a paediatric palliative care setting (*n* = 16); participants were not registered nurses or nursing students (*n* = 4); and studies did not focus on spirituality or spiritual care as a primary focus (*n* = 5). A hand search did not uncover any additional records. Ultimately, ten studies were included in the final synthesis.

**Figure 1:**
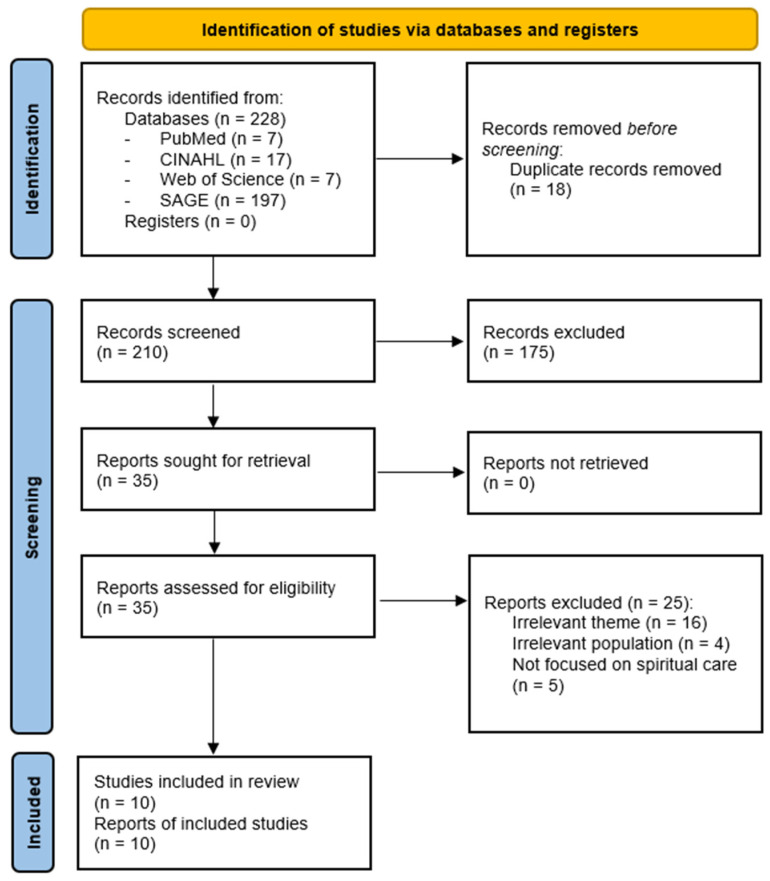
Flow diagram of the process for searching and selecting studies.

Pairwise percentage agreement and Cohen’s kappa were calculated before consensus discussions for the title and abstract screening stage. Percentage agreement ranged from 83.2% to 92.1%, and Cohen’s kappa ranged from 0.65 to 0.79, indicating substantial agreement according to the Landis and Koch [29] criteria. Inter-rater reliability was calculated only for the title and abstract screening stage, as full-text eligibility decisions were finalised through consensus by the entire author team. All included studies achieved a minimum JBI quality appraisal score of 70%. The study selection process is summarised in the PRISMA 2020 flow diagram (Figure 1).

### 3.2. Characteristics of Included Studies

The ten included studies were conducted across six countries: the United States of America (*n* = 5), Great Britain (*n* = 1), Malaysia (*n* = 1), Switzerland (*n* = 1), Brazil (*n* = 1), and Oman (*n* = 1). The combined sample comprised 237 nurses, 26 children, and 57 family members. Of these, the 26 children and 57 family members were direct participants in a single study (Scott et al. [30]). All remaining studies recruited nurses as the sole participant group, and any references to children’s or families’ perspectives were reported indirectly through nurse accounts. Although the eligibility criteria permitted the inclusion of nursing students, none of the 10 included studies recruited nursing students; all study samples consisted exclusively of registered or licenced nurses with active clinical experience in paediatric palliative or end-of-life care settings. The implications of this for pre-registration education are discussed in Section 4.2. Nine studies utilised qualitative methodological designs, including phenomenological, interpretive phenomenological, descriptive qualitative, and case study approaches. In contrast, one study employed a descriptive correlational (cross-sectional) quantitative design. Study participants were primarily nurses working in paediatric oncology, paediatric intensive care, community palliative care, and end-of-life care settings. The characteristics of the included studies are presented in Table 1.

**Table 1:** Characteristics of the included studies.

Author, Year, Country	Study Design	Objectives	Care Setting	Primary Focus	Sample (*n*)	Main Findings
Bergsträsser et al. [31], 2017; Switzerland	Qualitative, phenomenological	Describe the experiences and needs of health workers in paediatric palliative care.	Hospital and community (mixed)	No	*n* = 18 paediatric nurses *n* = 6 visiting nurses	All participants understood paediatric palliative care to be an integral part of their professional duty and a highly valuable aspect of their work. They described end-of-life care as very challenging, as it required a lot of emotional involvement (depending on the child’s medical condition and the emotional struggles the families had gone through). Most participants in the study reported that they were not sufficiently prepared to undertake such a demanding task, and many lacked specific training in paediatric palliative care. Nurses also recognised the importance of addressing families’ existential and spiritual needs, though many reported feeling insufficiently prepared to do so.
Chong and Abdullah [32], 2017; Malaysia	Qualitative, phenomenological	To research the experiences of visiting nurses in palliative care when caring for children at home.	Community/home-based	No	*n* = 16 visiting nurses	Content analysis identified 2 categories: challenges faced by nurses and coping strategies. The topics were: communication challenges, inadequate knowledge, personal suffering, system challenges, coping skills, interpersonal coping strategies and system supports.
Conner and Uddin [27], 2016; United States of America	Quantitative, descriptive correlational	To determine whether characteristics of nurses, level of comfort in caring for the dying, and spirituality are associated with the time of child referral to paediatric palliative care.	Hospital (multiple settings)	No	*n* = 105 nurses	Certain characteristics of nurses (age, work unit, nursing work experience, and palliative care education) predicted when they would refer a child to paediatric palliative care. Spirituality and comfort of nurses in caring for a dying child were not associated with referral of the child to palliative care. The main predictors of a child’s referral to palliative care were the nurse’s age and her work experience.
Ferrell et al. [33], 2016; United States of America	Qualitative, descriptive research	Research experience of nurses in communicating with children about spirituality.	Paediatric oncology (hospital)	Yes	*n* = 30 nurses	Nurses’ conversations about spirituality with children revealed that children questioned about God and the reasons for their illness, wanted to talk about the afterlife as a way of understanding their limited lifespan, and shared descriptions of the afterlife (described as heaven). Nurses conveyed the importance of being present and involved in spiritual communication with children. Nurses emphasised the need to draw on their own personal spirituality and life experiences to engage meaningfully in spiritual communication with dying children.
Hendricks-Ferguson et al. [34], 2015; United States of America	Qualitative, descriptive research	To research the experiences of nurses and their perspectives on communication about palliative care.	Paediatric oncology (hospital)	No	*n* = 16 nurses	Nurses’ experiences were divided into six themes. The six themes identified were: trust in caring, lack of communication skills, difficulty initiating palliative discussions, experiences with a child’s first death, importance of mentoring, and being present with an open heart. They believed they lacked sufficient communication skills to engage in open conversations. It was difficult to initiate palliative care discussions with dying children (struggling with unknown). They also discussed their first experiences when they witnessed the death of a child. They emphasised the importance of mentoring in communication during the experience of the first death of a child. They thought about how to be present with an open heart.
Kobler et al. [35], 2020; United States of America	Qualitative, case study	Research experiences of health workers during early paediatric palliative care.	Children’s Hospital (inpatient)	No	*n* = 6 nurses	Four outcome themes were identified: professional responsibility, staying connected, grounded uncertainty, and holding in—reflecting the emotional and relational complexity of early end-of-life awareness. The experiences of individual healthcare professionals were grouped into three dimensions: internal, relational, and awareness. Healthcare professionals sought to fulfil the child’s needs comprehensively and to guide parents in the early stages of joint decision-making, helping them maintain hope and connection in their relationship with their child.
Nascimento et al. [36], 2016; Brazil	Qualitative, phenomenological	To describe the meaning of spirituality as perceived by nurses.	Paediatric ICU (PICU)	Yes	*n* = 11 nurses	Nurses recognised the importance and value of spiritual care. They were aware of the importance of spiritual needs. In practice, they tended to pay significantly more attention to physical needs than to spiritual needs. The absence of spiritual care was attributed by nurses to a lack of time, with the child’s age and level of consciousness also being contributing factors.
Judith Roach et al. [37], 2023; Oman	Qualitative, phenomenological	To show the challenges faced by nurses in providing paediatric palliative care.	Paediatric haematology–oncology	No	*n* = 11 nurses	Three main themes: personal, educational and organisational challenges were the elements in the implementation of paediatric palliative care that nurses encountered in providing palliative care to children with leukaemia. Nurses faced personal challenges despite their education; they felt the need to upgrade their knowledge. Organisational challenges included the absence of dedicated spaces for prayer and family support, and insufficient access to psychological professionals to address spiritual and psychosocial needs.
Sawin et al. [38], 2019; United States of America	Qualitative, phenomenological	To describe the experiences and attitudes of paediatric oncology nurse managers regarding palliative care and communication at the end of a child’s life.	Paediatric oncology (hospital)	No	*n* = 11 nurses	The experiences of the managers, even 15 years ago (when they were beginning), vividly described their encounters with the death of a child. There was a feeling of insecurity, unpreparedness and lack of education. The overarching theme was ‘Fostering a Caring Climate’. Three themes: initial experiences and emotions of grief, family-centred care, and fostering a competent and caring workforce.
Scott et al. [30], 2023; United Kingdom of Great Britain and Northern Ireland	Qualitative, descriptive research	To recognise the spiritual needs of children with the help of their own experiences, parents and health workers.	Mixed paediatric palliative care	Yes	*n* = 7 nurses *n* = 26 children *n* = 57 family members	Topics: personal values (living life to the fullest), concerns (meaning of life), uncertainty about the future, determination to survive, accepting the future and struggle, and the role of religion.

### 3.3. Critical Appraisal Results

Critical appraisal scores for the nine qualitative studies ranged from moderate to excellent (Table 2). Two studies received a moderate-quality rating [33,38], while seven received a high-quality rating [30,31,32,34,35,36,37]. The quantitative study by Conner and Uddin [27] received a high-quality rating. Across the qualitative studies, the most consistent methodological weakness was insufficient reporting of researcher positionality (JBI criterion Q6), with six of the nine qualitative studies receiving a ‘No’ rating on this criterion [30,31,32,34,35,36,37]. A related shortcoming—failure to address the influence of the researcher on the research process and vice versa (Q7)—was noted in five studies [30,31,32,33,36,38]. These limitations are characteristic of earlier qualitative nursing research and reflect evolving standards for reflexivity reporting rather than fundamental design flaws. All other criteria, including congruity between the methodology and the research question, data collection, analysis, and ethical conduct of the research, were met by the majority of included studies.

**Table 2:** Critical appraisal results.

Included Paper (*n* = 10)	Method	Question No.										Quality Appraisal
		Q1	Q2	Q3	Q4	Q5	Q6	Q7	Q8	Q9	Q10	
Bergsträsser et al. [31]	Qualitative research	Y	Y	Y	Y	Y	N	N	Y	Y	Y	8/10 (80%) high quality
Chong and Abdullah [32]	Qualitative research	Y	Y	Y	Y	Y	N	N	Y	Y	Y	8/10 (80%) high quality
Ferrell et al. [33]	Qualitative research	Y	Y	Y	Y	Y	N	N	Y	N	Y	7/10 (70%) moderate quality
Hendricks-Ferguson et al. [34]	Qualitative research	Y	Y	Y	Y	Y	N	Y	Y	Y	Y	9/10 (90%) excellent quality
Kobler et al. [35]	Qualitative research	Y	Y	Y	Y	Y	U	Y	Y	Y	Y	9/10 (90%) excellent quality
Nascimento et al. [36]	Qualitative research	Y	Y	Y	Y	Y	N	N	Y	Y	Y	8/10 (80%) high quality
Judith Roach et al. [37]	Qualitative research	Y	Y	Y	Y	Y	N	Y	Y	Y	Y	9/10 (90%) excellent quality
Sawin et al. [38]	Qualitative research	Y	Y	Y	Y	Y	N	N	Y	N	Y	7/10 (70%) moderate quality
Scott et al. [30]	Qualitative research	Y	Y	Y	Y	Y	Y	N	Y	Y	Y	9/10 (90%) excellent quality
Conner and Uddin [27]	Cross-sectional study	Y	Y	Y	Y	Y	N	Y	Y	―	―	7/8 (87.5%) high quality

Note: Y = Yes; N = No; U = Unclear; ― = Not applicable; ≥90% = Excellent quality; 80–89% = High quality; 70–79% = Moderate quality; <70% = Low quality. Cross-sectional study: 1. Were the criteria for inclusion in the sample clearly defined? 2. Were the study subjects and the setting described in detail? 3. Was the exposure measured validly and reliably? 4. Were objective, standard criteria used to measure the condition? 5. Were confounding factors identified? 6. Were strategies to deal with confounding factors stated? 7. Were the outcomes measured validly and reliably? 8. Was an appropriate statistical analysis used? Qualitative research: 1. Is there congruity between the stated philosophical perspective and the research methodology? 2. Is there congruity between the research methodology and the research question or objectives? 3. Is there congruity between the research methodology and the methods used to collect data? 4. Is there congruity between the research methodology and the representation and analysis of data? 5. Is there congruity between the research methodology and the interpretation of results? 6. Is there a statement locating the researcher culturally or theoretically? 7. Is the researcher’s influence on the research, and vice versa, addressed? 8. Are participants and their voices adequately represented? 9. Is the research ethical according to current criteria, or, for recent studies, is there evidence of ethical approval by an appropriate body? 10. Do the conclusions drawn in the research report flow from the analysis or interpretation of the data?

### 3.4. Thematic Synthesis: Nurses’ Engagement with Spirituality in Paediatric Palliative Care

**Figure 2:**
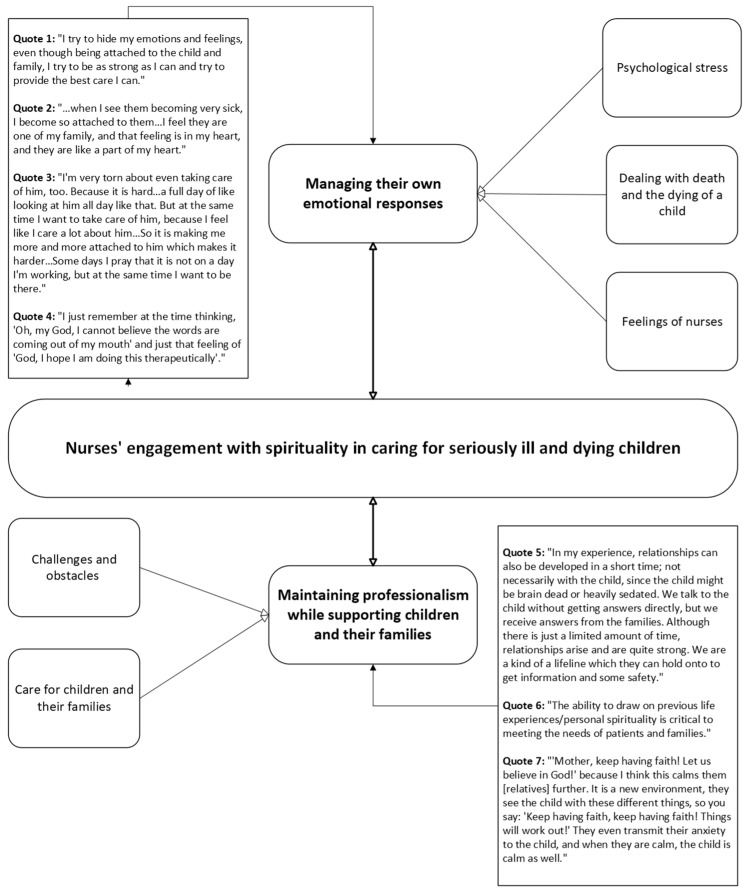
Synthesis of the included studies [29,31,34,35,36].

A comprehensive thematic synthesis of the ten included studies revealed one overarching theme: nurses’ engagement with spirituality in caring for seriously ill and dying children. During the analytical process, several additional candidate themes emerged, including nurses’ use of personal spirituality and faith as coping resources, interprofessional collaboration in spiritual care, and the role of organisational factors in facilitating or constraining spiritual care provision. Following iterative review and discussion among the author team, these candidate themes were not retained as independent analytical themes, as they were judged to be better conceptualised as contextual factors or sub-components of the two primary sub-themes rather than as distinct dimensions of nurses’ experiences. The decision to retain two sub-themes was made in the interests of analytical parsimony and conceptual clarity and was reached by consensus among all four authors. This main theme is supported by two sub-themes: (1) managing their own emotional responses, and (2) maintaining professionalism while supporting children and their families. These themes are illustrated in Figure 2, which depicts the hierarchical structure of the thematic framework: the overarching theme of nurses’ engagement with spirituality is shown as the central organising concept, with the two sub-themes representing the dual dimensions of nurses’ experiences—their internal emotional world and their external professional conduct.

#### 3.4.1. Sub-Theme 1: Managing Their Own Emotional Responses

Across the included studies, nurses consistently reported a substantial emotional and psychological burden stemming from caring for seriously ill and dying children. Feelings of discomfort, guilt, sadness, and helplessness were commonly described when supporting children with life-limiting conditions [32,34,35,39]. These emotional responses were strongly linked to a perceived lack of confidence in initiating or navigating difficult conversations with children or family members [38] and to uncertainty about whether all members of the healthcare team engaged in honest and open dialogue with children about their prognosis [35].

Nurses frequently described forming strong emotional bonds with children [34], often characterised by compassion and protective concern [34,37]. At the same time, some nurses reported withdrawing emotionally in intense situations [27], suggesting a coping strategy to limit exposure to distress. Despite these strategies, nurses often remembered moments of deep powerlessness, particularly when they struggled to find the right words or effectively support the parents of dying children [38].

Managing emotional responses emerged as a recurring challenge. Nurses reported difficulty regulating their emotional reactions, which contributed to cumulative burden and distress [37]. The concurrent experience of sadness, fatigue, worry, helplessness, nervousness, uncertainty, and guilt generated significant stress and could evolve into moral distress and professional isolation [32,35,37]. Suppressing negative emotions appeared particularly detrimental, being associated with psychological distress, anxiety, and depressive symptoms [37]. Encounters with dying children also prompted deep personal reflection, including thoughts about nurses’ own mortality [35]. Experiences of a child’s death, especially those in the home, were described as formative and significant for professionals [38].

Following a child’s death, nurses expressed a clear need for private space and time to grieve; when this need was unmet, unresolved grief was associated with emotional overload and, in some cases, anger [38]. Nurses thus experienced a persistent tension between their desire to remain emotionally connected to the child and their need to protect themselves from the intense demands of PPC [35]. Advocacy for enabling children to die at home, surrounded by family, was also evident, reflecting nurses’ commitment to preserving dignity and relational continuity at the end of life [38].

Sub-theme 1 (managing their own emotional responses) was supported by eight studies [27,30,31,32,33,34,36,37]. Sub-theme 2 (maintaining professionalism while supporting children and their families) was supported by seven studies [30,31,33,34,35,36,38]. Six studies contributed to both sub-themes.

#### 3.4.2. Sub-Theme 2: Maintaining Professionalism When Supporting Children and Families

Despite the significant emotional demands described above, nurses across the included studies consistently viewed end-of-life care as a central and meaningful part of their professional role. They regarded it as their professional duty to ensure that every child received the highest possible level of care, regardless of the obstacles encountered [31,35]. Many emphasised the importance of holistic care that extended beyond the child to encompass the entire family, recognising this as integral to supporting family values and strengthening relational bonds [38].

Nurses acknowledged that although physical care was generally well addressed in PPC, a persistent gap remained in the provision of spiritual care [31,32,34,35,36,37]. Barriers to spiritual care were most often attributed to time constraints and the child’s age or level of consciousness [31]. Nurses recognised the importance of spiritual needs and expressed a sincere desire to address them more fully; however, they frequently reported insufficient skills, limited experience, and a lack of confidence in engaging in spiritual or existential discussions with children and families [33,37]. Hope emerged as a central concept in nurses’ relationships with families, with those reporting higher levels of hope also demonstrating greater comfort and perceived competence in caring for dying children [27,31].

Complementing these qualitative accounts, the sole quantitative study in this review [27] examined nurse-level predictors of the intention to refer a child to paediatric palliative care. Contrary to expectations, neither nurses’ spiritual well-being (measured via the SWB scale, including its religious and existential well-being subscales) nor their comfort with caring for dying children (measured via the FATCOD scale) was a significant predictor of referral behaviour. Significant predictors were instead related to nurse demographics and clinical context. Nurse age was associated with a higher intention to refer children with cancer (OR = 1.03, *p* = 0.046), with each 5-year increase in age associated with approximately 17% greater odds of referral. Unit type (NICU versus other units) was associated with referral for extreme prematurity (OR = 0.382, *p* = 0.010) and HIV (OR = 0.455, *p* = 0.029), with NICU nurses less likely to refer in both cases. Years of clinical experience predicted the timing of referral for extreme prematurity at diagnosis (OR = 0.965, *p* = 0.034), with greater experience associated with later referral [27]. This pattern suggests that spiritual orientation alone does not translate into clinical action and that accumulated professional experience and unit context may play a more influential role in shaping palliative care decision-making than individual spirituality. Taken together, the qualitative and quantitative evidence points to a consistent conclusion: formal education and structured clinical experience, rather than personal spirituality, are the foundations upon which competent spiritual care practice must be built.

Effective communication with parents, especially those with unrealistic expectations, as well as with children and adolescents, was identified as the most challenging aspect of end-of-life care [32,34]. Nurses reported anxiety about saying the wrong thing, feelings of inadequacy when discussing prognosis openly, and a general sense of unpreparedness when initiating palliative care conversations [34]. Communication about spirituality was perceived as one of the most demanding areas of practice [33]. Nevertheless, it was essential for establishing trust and enabling families to feel supported throughout the dying process [35].

Despite these challenges, nurses reported strategies to support children and families. These strategies included maintaining presence, actively listening, using creative and age-appropriate communication methods, and carefully attending to non-verbal cues, especially with younger children or those with limited verbal abilities [32,36]. Positive emotions such as gratitude, fulfilment, and satisfaction were reported, especially when nurses felt supported in collaborative team environments [34]. These findings suggest that, while spiritual care in PPC is associated with considerable strain, it is also experienced as a source of meaning and professional purpose.

Based on the GRADE-CERQual assessment (Table 3), moderate confidence was assigned to both synthesised findings. For Sub-theme 1, minor methodological limitations were noted (primarily incomplete reporting of researcher reflexivity in three studies), coherence was assessed as adequate given the consistent patterning of emotional burden across heterogeneous settings, and data adequacy was considered sufficient given the rich qualitative accounts across eight contributing studies. For Sub-theme 2, the same reasoning applied. Relevance to the review question was assessed as high, as all contributing studies directly addressed nurses’ professional role in the provision of spiritual and end-of-life care.

**Table 3:** GRADE-CERQual at the level of findings.

Synthesised Finding	No. of Studies Contributing	Methodological Limitations	Coherence	Adequacy	Relevance	CERQual Assessment
Sub-theme 1: Managing one’s own emotional responses	8	Minor concerns	No/minor concerns	Adequate	High relevance	⊕⊕⊕◯ Moderate
Sub-theme 2: Maintaining professionalism when supporting children and families	7	Minor concerns	No/minor concerns	Adequate	High relevance	⊕⊕⊕◯ Moderate

Note: GRADE-CERQual, Grading of Recommendations Assessment, Development and Evaluation–Confidence in the Evidence from Reviews of Qualitative Research. Confidence ratings are indicated as follows: ⊕⊕⊕◯ = moderate confidence. The symbol ⊕ indicates confidence in the evidence, whereas ◯ indicates a reduction in confidence.

## 4. Discussion

This systematic review reveals that nurses in paediatric palliative care occupy a uniquely demanding professional position, simultaneously navigating profound emotional vulnerability and a responsibility to provide holistic, spiritually sensitive care to dying children and their families. The thematic analysis identified a complex interplay between emotional burden, spiritual engagement, professional commitment, and systemic constraints. The main theme of the study is nurses’ engagement with spirituality while caring for seriously ill and dying children. This theme is supported by two sub-themes that highlight the dual aspects of nurses’ experiences: managing their own emotional responses and maintaining professionalism while supporting both children and their families. These findings offer important insights for clinical practice, education, and healthcare organisations.

The findings of this review are broadly consistent with, yet meaningfully extend, those of McConnell, Scott and Porter [17], whose mixed-method review synthesised healthcare staff experiences of providing end-of-life care to children across 35 studies. That review identified similar themes of emotional burden, communication difficulties, and insufficient preparation among the wider clinical workforce. However, it was not restricted to nurses, did not isolate spiritual care as a distinct domain of inquiry, and did not employ a formal thematic synthesis methodology. The present review advances this evidence base in three ways: by focusing exclusively on the nurse-specific perspective, by identifying spiritual care as the primary object of analysis rather than end-of-life care in general, and by applying the validated thematic synthesis approach of Thomas and Harden [26] to a body of studies published up to November 2025. These methodological distinctions allow for a more precise and actionable synthesis of the barriers and facilitators that shape nurses’ capacity to deliver spiritual care in paediatric palliative settings.

The discussion is organised around four key implications: for clinical practice, nursing education, organisational policy and workforce support, and future research.

### 4.1. Implications for Clinical Practice

The review highlights that nurses in PPC work under ongoing emotional and moral challenges. They often experience intense feelings such as sadness, fear, helplessness, and reduced well-being. This is particularly true when nurses relate to the parents of their patients, especially if their own children are of a similar age to those they are caring for [32,37,38]. This emotional proximity may intensify empathic engagement but simultaneously heightens vulnerability to spiritual and psychological distress. The finding that nurses nonetheless regard end-of-life care as a valuable and integral professional responsibility, despite such strain, reflects a strong moral commitment [31,35]. Without systematic support—including supervision, debriefing, and access to psychological resources—this commitment may become unsustainable. In the broader healthcare literature, a sustained lack of organisational support has been associated with burnout, moral distress, and staff attrition [40]. Although the included studies did not longitudinally track these outcomes in paediatric palliative nurses specifically, the emotional patterns documented—cumulative distress, unresolved grief, and professional isolation—are consistent with established preconditions described in the burnout literature.

The review further highlights that nurses’ spiritual distress is closely interwoven with the experience of providing spiritual care. Children with serious illnesses clearly have spiritual needs that require recognition and sensitive responses [30]. Nevertheless, nurses frequently feel underprepared to provide spiritual care and report that physical care continues to dominate over spiritual dimensions [36]. This gap between perceived need and perceived competence poses a significant challenge to holistic care provision, as reported by the majority of the included studies. Although some nurses express a genuine desire to address the spiritual dimensions of a child’s experience [35], they also report insufficient skills, limited confidence, and inadequate experience in engaging in spiritual and existential conversations with children and families [33,37]. These findings suggest that spiritual care in PPC remains largely dependent on individual motivation and informal learning, rather than on structured educational and organisational frameworks.

A notable conceptual limitation that warrants explicit discussion is the heterogeneity in how spirituality was defined and operationalised across the included studies. None of the ten studies provided an explicit, standardised definition of spirituality or distinguished it systematically from religiosity. Several studies appeared to conflate the two constructs, most notably those that discussed spiritual care primarily in terms of prayer, chaplaincy referral, or engagement with religious rituals [31,33,36]. This conflation risks narrowing the scope of spiritual care to religious practice alone, thereby excluding the broader existential and meaning-centred dimensions of spirituality that are particularly salient for children and families who do not identify with a formal religion. The absence of a shared conceptual framework across studies limits the comparability of findings and may partly explain the persistent ambiguity nurses report when attempting to identify and respond to spiritual needs in clinical practice. To address this in future research and clinical education, we recommend adopting validated, nurse-applicable spiritual assessment tools that operationalise spirituality in an inclusive, non-religious manner. Neither FICA nor HOPE was identified in the included studies; they are proposed here based on their established use in the adult palliative care literature [41,42]. Two such tools are the FICA Spiritual History Tool [42] and the HOPE Questions model [41], both of which guide structured spiritual conversations without presupposing religious belief. Similarly, the EPICC Spiritual Care Competency Self-Assessment Tool [10], although a validated instrument for assessing spiritual care competency in nurses and midwives, was developed and tested primarily in adult acute care and general clinical education settings. Its applicability to nurses working specifically in paediatric palliative care has not yet been established, and adapting it for this population is a further research priority. Adapting FICA/HOPE or EPICC and formally validating them specifically for paediatric palliative nursing settings represent an important research priority that the current evidence base does not yet address.

Communication emerged as a central theme and a critical determinant of the quality of spiritual care. Sensitive, honest, and clear communication is essential for identifying spiritual needs, enabling children and families to share their feelings and experiences, and supporting meaning-making in the face of serious illness [31,36]. Communicating with parents, especially those with unrealistic expectations, and with adolescents who may show unexpected emotional reactions, is particularly challenging [32,34]. Nurses report anxiety about initiating such conversations, fear of saying the wrong thing, and a sense of inadequacy in open discussions about prognosis and end-of-life matters [17,34,37]. Spiritual communication is perceived as one of the most demanding areas of palliative nursing practice [33], yet it is essential for establishing the trust necessary for effective end-of-life care [35]. These findings emphasise the need to prioritise communication training, particularly in areas such as spiritual assessment, managing unrealistic expectations, and responding sensitively to strong emotions within PPC education and professional development.

A further dimension that the included studies addressed only superficially is the developmental variability in children’s understanding of death and spiritual experience. Spiritual needs and the capacity to articulate existential concerns differ substantially across paediatric age groups, and this heterogeneity poses distinct challenges for nurses’ communication strategies. Infants and toddlers lack a conceptual understanding of death and are primarily responsive to physical comfort, relational presence, and sensory continuity. Preschool-aged children typically hold magical or reversible conceptions of death and may express spiritual distress through play, symbolic language, or behavioural change rather than through verbal disclosure. School-aged children progressively develop an understanding of death’s permanence and universality. They may pose direct questions about the afterlife, the meaning of suffering, or the existence of God, as indeed was reported by nurses in the study by Ferrell, Wittenberg, Battista and Walker [33]. Adolescents, by contrast, grapple with existential questions in a manner increasingly similar to adults, including concerns about identity, legacy, and the fear of being forgotten, but may be more reluctant to disclose these concerns to healthcare providers. Nurses in paediatric palliative care must therefore be equipped not merely with generic spiritual care competencies but with developmentally sensitive communication skills adapted to each stage of childhood. Incorporating child development frameworks into spiritual care education—alongside communication strategies for non-verbal or cognitively impaired children—represents a critical and largely unaddressed training need.

The strategies identified in the included studies—such as maintaining presence, using creative and age-appropriate communication methods, and practising active listening—provide practical frameworks for enhancing both communication and spiritual support [32]. Careful attention to non-verbal communication is highlighted as particularly valuable in assessing spiritual needs in younger children or those with limited verbal ability [36]. These approaches are consistent with holistic models of PPC and reinforce the view that spirituality in this context encompasses meaning, dignity, and relational connectedness.

The findings also illuminate the range of coping mechanisms employed by nurses in PPC. Some nurses emphasise the importance of maintaining professional boundaries as a strategy for managing emotional exposure [32]. In contrast, others report experiencing extreme anxiety and a sense of abandonment when faced with staff shortages and inadequate organisational support [37]. These variations indicate that individual resilience alone is insufficient; structural and institutional interventions are essential. Ensuring adequate staffing ratios, promoting access to psychological and spiritual support for nurses, and formally recognising bereavement needs following a child’s death are measures likely to improve job satisfaction and support staff retention in both hospital and community paediatric palliative settings [34,38].

The findings also raise important questions about the ethical boundaries of the nursing role in spiritual care and the influence of nurses’ own spiritual or religious beliefs on their practice. None of the included studies explicitly addressed when spiritual care should be transferred to a specialist—such as a hospital chaplain or a community spiritual care provider—rather than remaining within the nurse’s scope of practice. This represents a significant gap, as the absence of clear interprofessional boundaries may leave nurses uncertain about their responsibilities and inadvertently expose them to ethical discomfort when spiritual conversations exceed their training or personal capacity. Establishing structured referral pathways and formalising collaboration with chaplaincy services within paediatric palliative care teams would help clarify these boundaries and ensure that children and families receive appropriately specialised spiritual support. Equally, the influence of nurses’ own spiritual beliefs on their practice received no systematic attention across the included studies. Evidence from the broader nursing literature suggests that nurses with strong personal religious affiliations may, consciously or otherwise, impose their own frameworks when responding to patients’ spiritual needs. In contrast, those with no spiritual orientation may avoid the domain altogether [43]. Fostering reflexivity—the capacity to critically examine how one’s own values and beliefs shape professional behaviour—should therefore be an explicit component of spiritual care competency frameworks in both undergraduate curricula and continuing professional development. Structured spiritual care assessment in paediatric settings should also consider the perspectives of children and families. Selman et al. [44] documented spiritual care needs across nine countries from the patient and caregiver perspective, highlighting significant unmet needs that health professionals—including nurses—are often ill-equipped to address. Similarly, Wiener et al. [45] identified practical tools for implementing psychosocial and spiritual care standards in paediatric oncology, offering a structured assessment model that could be adapted for broader paediatric palliative care contexts.

### 4.2. Implications for Nursing Education

In terms of education, the review identifies a pronounced need for enhanced preparation in PPC and spiritual care. Nurses consistently report gaps in their knowledge of the dying process in children and emphasise the value of continuing professional development in this area [30,35,37,38]. Targeted educational interventions aimed at building confidence in spiritual assessment and communication could improve both the quality of care delivered and nurses’ own occupational well-being [30,35,37]. Given projections suggesting growth in the number of children requiring PPC in the coming decades [5], investment in pre- and post-registration education and institutional support is increasingly indicated—though the strength of this recommendation should be tempered by the small and predominantly qualitative evidence base of the current review.

### 4.3. Implications for Organisational Policy and Workforce Support

At a systems level, the review underscores the importance of legislation, shared best practice, and international collaboration in advancing PPC worldwide [46]. Establishing evidence-based guidelines that incorporate various aspects of care identified in this review—such as spiritual assessment, family-centred communication, staff support, and bereavement care—would create a structured framework for clinical practice. These guidelines could be integrated into current national and international paediatric palliative care guidelines [47]. Such guidelines could help reduce practice variability and promote greater consistency in spiritually sensitive, holistic care.

The findings carry important implications for healthcare leadership and nursing management. Senior leaders in paediatric palliative care settings should be aware of the emotional and spiritual dimensions of nurses’ professional experiences and actively support organisational cultures that validate them. Regular team discussions, clinical supervision, reflective practice groups, and structured psychosocial support interventions may mitigate emotional distress and reduce professional isolation among nurses in PPC. Among evidence-based interventions demonstrated to reduce emotional burden among healthcare professionals in high-intensity clinical settings, Schwartz Rounds merit particular attention. Originally developed at the Massachusetts General Hospital and evaluated by Lown and Manning [48] in an adult general hospital setting, Schwartz Rounds provide a structured, multidisciplinary forum for clinicians to reflect openly on the emotional and social dimensions of patient care. Lown and Manning [48] reported associations between participation and reduced feelings of isolation, improved team communication, and greater capacity for compassionate engagement. It should be noted, however, that this evaluation was conducted exclusively in an adult hospital context, and no study included in the present review evaluated Schwartz Rounds specifically in paediatric palliative care or in relation to spiritual care outcomes. Their inclusion here is therefore based on transferability from a related field; formal evaluation of their effectiveness and acceptability in paediatric palliative nursing settings is recommended before broad implementation. A second well-supported approach is structured reflective supervision, in which nurses engage in facilitated one-to-one or small-group reflection on emotionally demanding clinical encounters. Unlike informal debriefing, structured supervision involves a trained facilitator and a defined reflective framework, ensuring that emotional processing is systematic rather than incidental. Paediatric palliative care teams should consider piloting both interventions and evaluating their acceptability and effectiveness in this specific context, with protected time and dedicated facilitation, as components of a broader staff well-being strategy. Providing structured opportunities for open dialogue at the ward level is essential for collective coping and sustaining a compassionate, competent nursing workforce.

### 4.4. Strengths and Limitations

A key strength of this review is its adherence to PRISMA 2020 guidelines [18] and the use of the JBI critical appraisal framework, which ensured methodological rigour and transparency [19]. The multi-national scope of the included studies enhances the breadth of the synthesis, and the use of thematic synthesis following a validated method [26] supports the credibility of the identified themes. The prospective registration of the review protocol in the OSF Registries prior to data extraction further supports the transparency of the analytical process.

This review has several limitations that should be considered when interpreting its findings. First, restricting the search to English-language publications may have introduced language bias. This limitation is particularly consequential for a review of spiritual care, as spirituality, religious expression, and end-of-life meaning-making are strongly shaped by language, culture, and local religious traditions. Studies from non-English-speaking countries—including those examining spiritual care within Catholic, Islamic, Buddhist, or indigenous spiritual frameworks—may have been excluded, limiting the cultural breadth of the synthesis. Additionally, thesis databases (including ProQuest Dissertations & Theses Global and EThOS) and conference proceedings were not systematically searched, which may have further contributed to publication bias by underrepresenting null, inconclusive, or negative findings. Furthermore, the search did not include major databases such as Scopus, Embase, and PsycINFO, which may have further limited coverage of relevant studies, particularly those published in psychology, psychiatry, and the broader biomedical literature. Second, nine of the ten included studies were qualitative in design, with only one quantitative study meeting the inclusion criteria; the evidence base is therefore primarily experiential and perceptual, and the findings cannot be statistically generalised. Third, most of the included studies were conducted in high-income, predominantly English-speaking countries, especially the United States. This concentration limits the applicability of the findings to low- and middle-income countries, where paediatric palliative care access, nursing roles, religious structures, and institutional support differ substantially. Fourth, although the JBI checklists were applied rigorously, the imposition of a 70% minimum quality threshold may still permit the inclusion of studies with methodological limitations while simultaneously excluding studies with relevant experiential insights that scored marginally below this threshold. No sensitivity analysis was conducted to examine whether the thematic findings would change if studies rated at exactly 70% [32,37] were excluded; future syntheses should include such analyses as a standard quality check. Fifth, thematic synthesis was conducted from the findings sections of the included studies rather than from verbatim participant quotations, thereby introducing an additional layer of interpretive mediation by the primary authors. This is an acknowledged methodological limitation of thematic synthesis as a secondary analytical approach. Sixth, the review focused on nurses’ perspectives and did not systematically incorporate the views of children, families, chaplains, or other members of the multidisciplinary team. Spiritual care in paediatric palliative settings is inherently interprofessional, and its full complexity may not be captured through the nurse perspective alone. One included study [30] employed a multi-informant design encompassing nurses, children, and family members. However, nurse data were extracted separately and prioritised in the synthesis; it cannot be excluded that the mixed-participant design introduced subtle interpretive influences on the thematic findings. The decision to include this study was reached by consensus among all authors and represents a minor deviation from the predefined eligibility criteria.

### 4.5. Future Research

Future research should employ longitudinal and cross-cultural study designs to examine the long-term effects of targeted educational and support interventions—including structured spiritual care training, reflective supervision, and Schwartz Rounds—on both nurse well-being and the quality of spiritual care experienced by children and families. Studies conducted in low- and middle-income countries are particularly needed, given that these settings bear the greatest burden of paediatric palliative need yet are substantially underrepresented in the existing evidence base. The creation and validation of culturally adapted, nurse-specific spiritual care competency frameworks, along with their incorporation into PPC curricula, should be prioritised.

Future research should also include the perspectives of children, families, chaplains, psychologists, and other members of the multidisciplinary team. Although this review focused on nurses, spiritual care in paediatric palliative care is inherently interprofessional, and a more complete evidence base requires synthesis across the full care team. Studies should additionally examine how spiritual care needs and communication strategies differ across paediatric developmental stages—including infants, young children, school-aged children, adolescents, and children with cognitive or communication impairments—as this developmental dimension was largely absent from the included studies.

The development and psychometric validation of culturally adapted spiritual care assessment tools specifically for paediatric palliative settings—particularly in non-English-speaking and low-resource contexts—represent a further research priority. Finally, studies examining the prevalence and severity of spiritual distress among nurses in paediatric palliative care, using validated outcome measures (such as burnout inventories, moral distress scales, and compassion fatigue instruments), would strengthen the empirical basis for organisational support recommendations that the current qualitative evidence base can only suggest.

## 5. Conclusions

This systematic review synthesised evidence from ten qualitative and cross-sectional studies examining nurses’ experiences with spiritual care in paediatric palliative care across six countries. The synthesis revealed one main theme: nurses’ engagement with spirituality while caring for seriously ill and dying children. This theme is supported by two sub-themes: managing their emotional responses and maintaining professionalism in their support for children and families. The findings demonstrate that nurses in PPC experience substantial emotional, psychological, and existential strain and frequently feel underprepared to provide spiritual care or to navigate spiritually charged conversations with dying children and their families. Despite these challenges, nurses consistently regard end-of-life care as a central and meaningful dimension of their professional role. A persistent gap between the recognition of spiritual care as important and the actual capacity to deliver it was identified across the included studies, associated with insufficient education, limited institutional support, and systemic barriers. These findings underscore the need for structured educational programmes in spiritual care competency, dedicated psychosocial support mechanisms for nursing staff, and evidence-based organisational policies in paediatric palliative care. Future research should prioritise longitudinal, culturally diverse designs to evaluate the effectiveness of targeted interventions on both nurse well-being and the quality of spiritual care experienced by children and families at the end of life. The conceptual heterogeneity in definitions of spirituality across the included studies further underscores the need for consensus on standardised, inclusive frameworks before the evidence base can be meaningfully extended.

## Data Availability

The datasets generated and analysed in this review are not publicly available but can be provided on reasonable request. Materials available upon request include standardised data extraction forms, completed JBI critical appraisal sheets for all included studies, the thematic coding scheme and free codes generated during thematic synthesis, and the PRISMA 2020 checklist. Requests should be directed to the corresponding author.

## Supplementary Materials

The following supporting information can be downloaded at: https://www.mdpi.com/article/10.3390/healthcare14131994/s1, Supplementary Material S1: Database-Specific Search Strings.

## Abbreviations

The following abbreviations are used in this manuscript:

PPC	Paediatric palliative care
PC	Palliative care
