# Supplementary material for: Nurses’ Experiences with Spiritual Care in Paediatric Palliative Care: A Systematic Review
Source: Healthcare (Basel). 2026 Jul 4;14(13):1994. doi: 10.3390/healthcare14131994 (PMC13361994; doi:10.3390/healthcare14131994)
Supplement: Supplementary file 1 [file healthcare-14-01994-s001.zip › healthcare-4354814-supplementary.pdf]

Supplementary Material S1

Database-Specific Search Strings

The following search strings were adapted for each bibliographic database in accordance with PRISMA 2020 reporting guidelines (Item 7). The core search concepts were: (1) nurses/nursing, (2) spiritual care/spirituality, (3) paediatric palliative care, and (4) child/adolescent population. Boolean operators (AND, OR) were applied consistently. Database-specific controlled vocabulary (MeSH terms for PubMed; CINAHL Subject Headings for CINAHL) was combined with free-text terms to maximise sensitivity. All searches were conducted in November 2025 and restricted to English-language publications.

1. PubMed (via MEDLINE)

Controlled vocabulary: MeSH (Medical Subject Headings). Date searched: November 2025. Records retrieved: n = 7.

```
(("Nurses"[MeSH Terms] OR "Nursing Staff"[MeSH Terms])
 OR (nurse[Title/Abstract] OR nurses[Title/Abstract] OR nursing[Title/Abstract]))
AND
(("Spirituality"[MeSH Terms] OR "Pastoral Care"[MeSH Terms])
 OR ("spiritual care"[Title/Abstract] OR spiritual*[Title/Abstract]))
AND
(("Palliative Care"[MeSH Terms] OR "Hospice Care"[MeSH Terms]
  OR "Terminal Care"[MeSH Terms])
 OR ("palliative care"[Title/Abstract] OR "end-of-life care"[Title/Abstract]
    OR "hospice care"[Title/Abstract]))
AND
(("Child"[MeSH Terms] OR "Adolescent"[MeSH Terms] OR "Infant"[MeSH Terms]
  OR "Pediatrics"[MeSH Terms])
 OR (child*[Title/Abstract] OR paediatric*[Title/Abstract]
    OR pediatric*[Title/Abstract] OR adolescent*[Title/Abstract]))
AND
(experience*[Title/Abstract] OR perception*[Title/Abstract]
 OR attitude*[Title/Abstract])
```

Filters: English language; all study types

2. CINAHL (via EBSCOhost)

Controlled vocabulary: CINAHL Subject Headings. Date searched: November 2025. Records retrieved: n = 17.

```
((MH "Nurses+" OR MH "Nursing Staff+")
 OR (TI nurse OR TI nurses OR AB nurse OR AB nurses))
AND
((MH "Spirituality" OR MH "Pastoral Care")
 OR (TI "spiritual care" OR AB "spiritual care"
   OR TI spiritual* OR AB spiritual*))
AND
((MH "Palliative Care+" OR MH "Hospice Care+" OR MH "Terminal Care")
 OR (TI "palliative care" OR AB "palliative care"
    OR TI "end-of-life care" OR AB "end-of-life care"))
AND
((MH "Child+" OR MH "Adolescence" OR MH "Pediatric Nursing")
 OR (TI child* OR AB child*
    OR TI paediatric* OR AB paediatric*
    OR TI pediatric* OR AB pediatric*))
AND
(TI experience* OR AB experience*
OR TI perception* OR AB perception*)
```

Limiters: English language; peer-reviewed

3. Web of Science (Core Collection)

No controlled vocabulary (keyword search only). Date searched: November 2025. Records retrieved: n = 7.

```
TS=((nurse OR nurses OR nursing)
   AND ("spiritual care" OR spiritual*)
   AND ("palliative care" OR "end-of-life care" OR "hospice care")
   AND (child* OR paediatric* OR pediatric* OR adolescent*)
   AND (experience* OR perception* OR attitude*)
```

Language: English
Document types: Article, Review

4. SAGE Journals

No controlled vocabulary (keyword search only). Date searched: November 2025. Records retrieved: n = 197.

```
("spiritual care" OR "spirituality")
AND (nurse OR nurses OR nursing)
AND ("palliative care" OR "end-of-life care" OR "hospice care")
AND (child* OR paediatric OR pediatric OR adolescent* OR youth)
```

Language filter: English

Note: SAGE Journals does not support MeSH or subject headings. The higher record count (n = 197) reflects the platform's broader keyword-matching behaviour compared to that of databases with controlled-vocabulary indexing.

Notes on Adaptation

The general search string reported in the main text of the manuscript represents a simplified, cross-database version for readability: (experience* OR perception) AND nurse AND (spiritual* OR "spiritual care") AND (child* OR "young adult*" OR adolescent*

OR youth) AND "paediatric palliative care". Each database-specific string above preserves this conceptual structure while incorporating appropriate syntax, field codes, and controlled vocabulary for that platform.
